# Confidence in Procedural Skills before and after a Two-Year Master's Programme in Family Medicine in Gezira State, Sudan

**DOI:** 10.1155/2017/6267015

**Published:** 2017-11-28

**Authors:** K. G. Mohamed, S. Hunskaar, S. H. Abdelrahman, E. M. Malik

**Affiliations:** ^1^Department of Family and Community Medicine, Taibah University, Medina, Saudi Arabia; ^2^Department of Global Public Health and Primary Care, University of Bergen, Bergen, Norway; ^3^Department of Family and Community Medicine, University of Gezira, Medani, Sudan; ^4^National Centre for Emergency Primary Health Care, Uni Research, Bergen, Norway; ^5^Department of Community Medicine, Faculty of Medicine, University of Khartoum, Khartoum, Sudan

## Abstract

Many postgraduate family medicine training programmes have been developed to meet the worldwide dire need for practicing family physicians. This study was conducted in Gezira state of Sudan in a “before-and-after” design in the period of 2010–2012 with the aim to assess improvements in candidates' confidence in performing certain clinical skills. A self-evaluation questionnaire was used with a five-grade scale (1–5) to assess candidates' confidence in performing 46 clinical skills. A group of 108 participants responded for both the “before” and the “after” questionnaire: the response rate was 91% (before) and 90% (after). In general, a positive progress trend was detected. The mean skill value for all skills was 3.23 (before) and 3.93 (after) with a mean increase of 21.7% (*P* < 0.001). Male students scored constantly higher than females both before and after completing the master's programme, while females showed a higher percentage in progress. Scores in certain medical disciplines were higher than others. However, disciplines with low scores in the beginning, such as psychiatry and ophthalmology, showed the highest progress percentage. The results show a significant increase in confidence in performing procedural skills designed in the curriculum of the GFMP master's programme.

## 1. Introduction

Clinical skills represent a major and important part in family medicine practice. Family physicians should master a variety of clinical competencies and should be prepared to work in rural areas without easy access to specialist services or advanced technology. Quality control of postgraduate family medicine training programmes should facilitate the physicians' role and ensure patient's safety. During and after training, it is important to assess the training outcome either objectively (trainee's competence) or subjectively (confidence) individually or as part of the outcome evaluation of family medicine training programmes. The accelerated rate of growth in medical knowledge represents a challenge in medical education [1, 2]. The inculcation and assessment of clinical skills are an important issue that needs more attention. Additional research is needed in the medical educational context, particularly in view of the continuous reformations of the medical curricula [3, 4].

There are several theories, models, and teaching strategies when it comes to the teaching and learning of clinical skills. Miller's pyramid [5], as well as its steps, “know,” “know how,” “show how,” and “do,” is simple and is widely used. Bloom's taxonomy including the cognitive, affective, and psychomotor domains has been historically used to classify and formulate the educational learning's objectives. It arranges the educational objectives in a hierarchy from less to more complex [6, 7]. The Dreyfus model of skills acquisition [8] is generally accepted in the clinical field with some modifications [9, 10]. It is widely used to describe developmental phases of skills acquisition, starting from the “novice” and “advanced beginner” to “competent” and “proficient” and ending as an “expert.”

There is only a small amount of literature on teaching and learning clinical skills and procedures in the field of family medicine related to such models and theories. Most curricula and programmes include lists of skills and procedures to be acquired by the students; evaluation studies of the results of such teaching strategies are scarce [11, 12]. Some institutions have published their lists of skills [13, 14]; however, competency in all domains is somewhat context-dependent, and economy, geography, disease spectrum, and epidemiology may play a role in selecting targeted skills.

In countries with advanced healthcare systems, strengthening of the system by qualified, well-trained family physicians leads to higher quality of healthcare services, more patient satisfaction, and better clinical outcomes [15]. In developing countries, various family medicine training programmes are established to fill the huge gap and to satisfy the need for skilled, qualified family physicians [16, 17]. Although family medicine specialization has been only recently emerging in Africa, research has shown that the role of skilled family physicians has been recognized by the African governmental and academic leaders as a positive asset [18, 19].

According to international reports, about 90% of the clinical situations at the community level are expected to be solved by family physicians at the primary care level [13, 20]. However, an extended list of skills may be required from African family physicians compared to areas with more developed clinical settings [21]. This is likely due to the contexts where African family physician practices, with more responsibilities, inadequate resources in healthcare facilities, long distances, and a poor referral and communication system with secondary hospitals. A WONCA consensus report on family medicine in Africa stated that the family physician must have a comprehensive set of skills which must be adapted to the local needs and resources [22].

The Gezira Family Medicine Project (GFMP), established in 2010 by the University of Gezira and the Gezira State Ministry of Health, Sudan, organized a two years' in-service master's programme in family medicine for medical doctors [23]. While the theoretical part of the curriculum was mainly provided as online lectures and seminars, practical clinical skills were mainly taught one day per week “at hospital training” and through the ongoing practice in the health centres. The GFMP model was described as “an inspiring example of family medicine training programme in Africa” in a consensus document of participants from 25 counties at the fifth annual PRIMAFAMED (Primary Care and Family Medicine Education Network) conference held in Victoria Falls, Zimbabwe, in 2012 [17]. The first batch of students of GFMP was included in an evaluation programme [23, 24] that also aimed to measure the training outcome, including confidence in the targeted clinical skills.

The aim of this paper is thus to assess in a “before-and-after” design the candidates' self-assessed confidence in performing both cognitive and psychomotor clinical skills included in the GFMP curriculum. In this study, clinical skills are defined as manual procedures and intellectual skills used in physical examination, history taking, diagnosis, and management. The study also evaluates demographic factors such as gender and age which affect confidence in clinical skills performance. It also compares the candidates' confidence and development in performing skills from different clinical disciplines.

## 2. Methods

### 2.1. Study Area

Gezira state lies mainly between the Blue Nile and the White Nile, in the central part of Sudan, south of Khartoum. It has a population of about 3.7 millions, relying mostly on agriculture and grazing; people reside mainly in about three thousand scattered small rural villages. Tropical diseases like malaria and bilharzias are endemic [25]. Noncommunicable diseases (NCDs) are following the regional emerging trend [25, 26], increasing the responsibility of an already weak healthcare system and sharing resources with traditional diseases.

The health system is composed of “healthcare centres” as the first line of primary care. Heath centres that differ in size and function are mainly served by nurses, “medical assistants,” and doctors (medical officers). “Rural hospitals,” the referral point from health centres, are led by medical officers. Many such hospitals have operating theatres and wards for inpatient care. Secondary care hospitals are found in cities and are served by specialist doctors. Tertiary care hospitals in Medani (state capital) represent the end referral point for the entire state and sometimes from neighbouring states. The secondary and tertiary hospitals are overburdened with patients who could have been managed at the primary care level, reflecting the need for qualified trained family physicians capable of providing higher-quality services with higher accessibility to the population.

### 2.2. The Gezira Family Medicine Project (GFMP)

Gezira state of Sudan had 115 medical officers (graduated doctors without postgraduate training) and no family medicine specialist at the start of 2010, reflecting a ratio of primary healthcare doctors to population of about 1 : 32,000. To bridge this huge gap, the Gezira Family Medicine Project (GFMP) was planned [23]. The GFMP master's curriculum was developed at the Faculty of Medicine, University of Gezira, by a committee including family physicians and representatives from the other disciplines (departments); national family medicine experts from other universities were also invited. The content of the curriculum took into consideration the national, regional, and international guidelines and principles of family medicine training. The compatibility of the future family physician with the health system and the locally needed set of skills was also considered.

The trainees of the GFMP were allocated to the health centres in both urban and rural areas. The GFMP curriculum aimed to train them to present both curative and preventive care in a continuous comprehensive manner. An “in-service” training programme is used at the GFMP. One day per week, the candidates visited one of the three major hospitals in Gezira (according to their location). Here, the candidates participated in clinical rounds, outpatient clinics, referral clinics, theatre, and so forth. One major objective for this activity was to learn the clinical and procedural skills required by the curriculum. Training methods used in the GFMP are described in detail in Table 2 in a previous paper [23].

It was decided that the curriculum should not include major surgical and obstetrical procedures like appendectomy, caesarean section, or management of ectopic pregnancy, as they are not part of GPs' clinical responsibilities in Gezira. These and other conditions needing major surgical intervention are referred to the nearby rural hospital and secondary or tertiary hospitals where relevant specialists in these disciplines or GPs with special training in such procedures are available.

Clinical manual procedures to be performed by the candidate were separately provided in a logbook that was distributed to all candidates at the start of the master's programme. The logbook is divided into sections according to the planned clinical rotations and includes a variety of clinical and practical skills. Teaching staff (hospital physicians) used to sign the candidates' logbooks, as part of their ongoing supervision; this normally occurs at their scheduled weekly hospital-based training. The signing attests to the skills the candidates observed or performed. Some skills needed to be signed more than once to confirm a higher level of competency. The logbook was recognized as a prerequisite to enter the final exam that included an essay, MCQs, and short and long OSCE cases. In addition to the hospital-based training, candidates also acquired clinical skills from their colleagues in the same health centre or through online communication with colleagues in other centres. Telemedicine communication with university professors was also used to support skills' development [24].

### 2.3. Study Population and Study Design

A before-and-after design is used in this study. Data for the study was collected at the start of the master's programme in November 2010 and after two years (at the end of the study period, just before the exam). The “before” data collection targeted 207 candidates (total number enrolled in the first batch of candidates in GFMP); 188 of them responded (91% response rate). The “after” data collection targeted 125 candidates who were still affiliated with the master's programme at its end in 2013; 113 of them responded (90% response rate). When we compared the same respondents “before and after,” only candidates who responded both “before” and “after” were included (108 candidates); candidates who did not respond to either the “before” or the “after” questionnaire were excluded in this comparison. The 108 respondents did not always answer all skill questions in both questionnaires; the range of the number of candidates responding to an individual skill was 82–108 candidates with a mean of 100 candidates; this could be automatically recognized by SPSS programme when using paired *t*-test to compare the two sets of responses.

### 2.4. Data Collection

The designed questionnaire for this study included a selected list of 46 clinical skills (shown in Table 1). The selected skills were from all medical disciplines included in the curriculum (medicine, surgery, obstetrics, etc.); they were also different in their level of difficulty. Certain skills, including some major surgical skills, were included in the questionnaire, although they were not a part of the curriculum. That was meant to assess if the candidates gained a lot of such skills at the end of the training programme, which indicates a deviation from the targeted learning objectives.

A five-grade scale was used, starting from “very confident,” followed by “confident,” “not fully confident,” “uncertain,” and finally “not able” to perform the skill. Consequently, the candidates were assigned values of 5, 4, 3, 2, or 1. The questionnaire was anonymous but had an identifiable code. Data collection was done at the start of the programme and after the two-year study period for the purpose of evaluating the candidates' progress.

Four other skills related to patient-education were assessed in the questionnaire: the use of insulin by diabetics, breast self-examination in women, smoking cessation, and feeding of malnourished children. A three-grade scale consisting of “yes,” “sometimes,” and “no” was adopted.

### 2.5. Statistical Analyses

Data were managed and statistically analysed using the SPSS® programme version 21. Results are presented as descriptive statistics with means, proportions, and percentages.

Regression analyses were performed to analyse whether personal background factors (age and gender), institutional factors (university graduation and locality), personal interest in family medicine, or clinical activity (reflected by number of electronic patient files opened by the candidate) affected the change in candidates' self-evaluation of own competence in clinical skills due to the master's programme.

Three different approaches were used for the analyses, all shown in Results. Firstly, to get the difference, we have simply subtracted the given skills' value before the master programme from the value chosen after the master program. Secondly, to remove the effect of initial scores, we computed residualized or regressed change scores. The procedure identifies cases where a person has changed more than would have been expected, based on the initial score. Regression analysis then is used to estimate (or predict) skill scores after the master's programme on basis of the correlation of skill scores after and before the programme. The predicted scores are then subtracted from the actual "after" scores. The remaining is the residual gain score, which means the amount of gain that is not due to the influence of the initial score. Thirdly, we calculated the amount of change as a percentage change. This was done by subtracting the "before" skill score from the "after" score, dividing the answer by the "before" score, and multiplying with 100 to convert this into ordinary percentage.

### 2.6. Ethical and Privacy Approvals

The ethical review committee at the Ministry of Health, Gezira state of Sudan, reviewed and approved the study on 8 March 2011. The study proposal was also approved by the Regional Committee for Medical and Health Research Ethics, Western Norway. The Norwegian Data Protection Official for Research also approved the privacy issues and patients' file management related to the scientific evaluation.

## 3. Results

For our study, a total of 108 candidates responded to the evaluation questionnaires both before and after the master's training programme; 46 (43%) of them were males, while 62 (57%) were females. This gender distribution is almost the reverse of the distribution recognized at the start of the master's programme (males: 57%; females: 43%). The total mean age at the start was 32.7 years; for males the mean age was 35.4 years (range: 25–59), while for females it was 30.7 years (range: 24–47).

Table 1 shows self-evaluation in performing selected clinical skills (*N* = 46 skills) categorized in disciplines. Results are shown as means, changes in mean, and *P* values. Scale values started from 5, “highest confidence,” to 1, “least confidence,” for each skill (*N* = 108 candidates). Large variation in candidates' self-evaluation performance is detected at the start of the programme, “before,” with a range of 1.72 to 4.78. Skills like measurement of blood pressure and abscess drainage scored highest at the start, “before,” while skills like cholesteatoma operation and eye fundoscopy got the lowest scores. Variation between the various medical disciplines ranged from 3.9 (minor surgery) to 2.33 (psychiatry). The total mean scoring for all skills was 3.23 at the start of the training programme.

Evaluation after the master's programme showed higher values in all 46 skills. Also it followed the same trend in individual skills variation and interdisciplinary variation. The increase was statistically significant for all skills except for tonsillitis management and uterine evacuation after abortion. The mean skill value after the programme for all skills was 3.93 with a mean difference of 0.70 from the “before” value, which represents a statistically significant difference (*P* < 0.001) with a progress percentage of 21.7%.

The mean skill value change had a range of 0.09–1.61. The most positive mean value change was recognized in skills like measuring visual acuity and management of major depression, while the least mean value change was recognized in skills like tonsillitis management and blood pressure measuring. The range of recorded confidence levels was from 1 to 5 (all categories used) for all skills and procedures, except four.

The 10 skills with lowest scores increased from 2.20 to 3.28, an increase of 49%, compared with a 22% increase for all skills. Among the 11 skills with lowest score change, 8 had a start level of more than 4.0 and the other 3 were major surgical procedures (Caesarean section, abortion, and appendectomy).

Table 1 also shows the mean scoring for the candidates in the different clinical disciplines. Candidates scored highest values in minor surgical skills and internal medicine both before and after the master's programme; however, the mean change value was highest in ophthalmology and psychiatry skills.

Table 2 shows the number of candidates who had least scoring (“unable” or “uncertain”) on performance of clinical skills before the programme and the change obtained during the training period. Skills with highest progress percentage are included (*N* = 23); the sequence is according to the progress percentage (*N* = 108). The change percentage represents the percentage of candidates who left this group of least scoring; the change was highest in skills like visual acuity measuring, ECG interpretation, and management of major depression.

The number of candidates decreased through the course of the master's programme from 207 at start to 125 at the end. The number of respondents to the first questionnaire amounted to 188 candidates. At the end of the master's programme, 108 candidates of the original 188 candidates (and of the 125 remaining) responded to the second questionnaire. To assess if there was a difference between the two groups on the baseline, we compared the group of 188 candidates who represent all respondents to the first questionnaire with the group of respondents who answered the second questionnaire (108 candidates) at the start of the programme. A total mean difference of 0.04 was observed between the two groups in all skills at the start of the programme. Although the difference is statistically significant (*P* < 0.001), it represents only a 1.2% difference in percentage compared with the difference between “before” (*N* = 108) and “after” (*N* = 108), which was 0.70, representing a 21.7% difference.

The candidates' competency to conduct certain patient-educational skills is shown in Table 3. Improvement in teaching mothers how to feed their malnourished children is represented by the highest mean value; however, there were statistically significant positive changes in all four assessed skills (*P* < 0.001).

Table 4 shows discipline-based, intergender variation. Males have constantly higher value in self-evaluation compared with females in all clinical disciplines both before and after assessment. The difference was statistically significant in all disciplines before the master's programme except for ophthalmology, whereas after the master's programme, the difference was not significant in internal medicine, laboratory skills, and ophthalmology.

**Figure 1:**
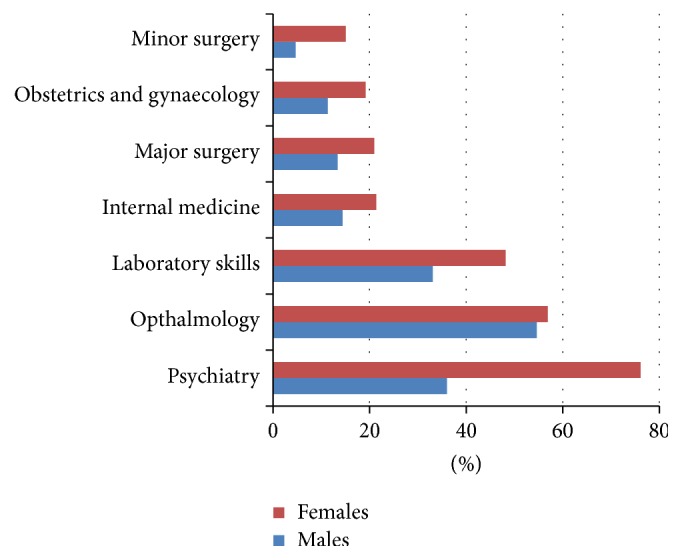
Gender variation in relative competency change in clinical disciplines among master's programme candidates of GFMP, Gezira state, Sudan (*N* = 108, 46 males and 62 females).

Figure 1 reflects the percentage change after the master's programme in medical disciplines (surgery, medicine, etc.) in relation to gender. Females showed higher score change in all skills compared with males. Such change was most prominent in psychiatry. Least development for both genders was in scores for minor surgery followed by obstetrics and gynaecology.

### 3.1. Regression Analyses for Explanatory Factors in Change of Skills Level

None of the three chosen approaches for analysing the change variable gave an overall statistically significant model (Table 5). The coefficients of determination (*R*^2^) were almost identical in the three analyses. However, we identified two single significant results. The doctors' interest in family medicine speciality after the master's programme turned out to have a positive and significant effect when residual gain score was used; that was performed while all other variables were controlled for in the analysis. The interpretation of the *B* coefficient is that one value increase on the doctors' interest increases the gain score by 0.21. When change was measured as percentage, gender had a significant (positive) effect, indicating that female doctors changed 12.4% more than male doctors; that was performed while all other variables were controlled for in the analysis.

## 4. Discussion

The trainees themselves perceived general progress in clinical skills competencies, according to their self-evaluation feedback. Interdisciplinary and gender variations were detected. The interdisciplinary variation in candidates' baseline skills (before the master's programme) can be attributed to the inadequacies in some undergraduate curriculum designs, which include giving relatively low weight to certain disciplines like psychiatry and ophthalmology. These disciplines scored least in the baseline and showed the highest positive change in percentage development after the master's programme. The in-service model of training could have been attributed to the outcome competency variations, giving a higher chance for competencies related to the candidates' clinical practice. In certain skills and disciplines, candidates have evaluated themselves so high in the baseline that there was not much room for further progress; examples include management of tonsillitis and postabortion evacuation, which showed no statistically significant change after the master's programme. However, this effect was controlled using regression analysis, and the lack of change was confirmed.

Male trainees evaluated their own skills as being better than those of females both before and after the master's programme. This trend is also recognized in other studies in different settings, where males show higher confidence in their own competencies [27]. On the other hand, female trainees could potentially show a higher progress percentage in all disciplines. However, this does not necessarily imply that females objectively gained more skills, since baseline self-evaluation by males could have affected males' progress percentage. Why males show higher confidence in self-evaluation in this study and other studies is not clear and needs further specific clarification. Nevertheless, one may speculate from this and other studies that males rate their confidence as higher than females for the same level of competence.

The positive progress in the patient education competencies reflects the role of the GFMP master's curriculum in adding the value of health promotion and preventive care to the medical practice of the trainees. This is an integral indicator of successful implementation of family medicine principles.

We performed three different regression analyses based on different measures of change; there are problems associated with all of them. The results by raw score or percentage of change show whether the persons have improved or not. However, persons having extreme scores initially (low or high) are met by ceiling or floor effects. Those with previous low scores have higher chances of improving, since there is more room (technically speaking) for improvement; those with high scores have greater chances of getting lower scores. Hence, people previously scoring in the middle range of the scale are more likely to experience changes, either negative or positive. To remove the effect of initial scores, we used residualized or regressed change scores, but the results came out with the same overall pattern.

Skills with low confidence scores at the baseline and skills with low change progress percent need to be given more attention both in undergraduate curricula and in family medicine postgraduate training. However, our results gave no grounds for concern about distribution or skewness in the acquisition of skills confidence based on starting level or small change in important procedures. On the contrary, skills with low positive change had a very high confidence level from the beginning. The only exceptions were the major surgical procedures, which had low priority in the curriculum.

### 4.1. Study Limitations

Firstly, the gradual decrease in the number of the trainees in the course of the training period can be regarded as a study limitation. However, this was managed by including candidates who responded to both the "before" and the "after" questionnaires in the study (108 candidates). A comparison of the results from the “before” questionnaire between the group of 188 candidates (all respondents before the dropout) and the group of 108 candidates included found only a minor difference at the starting point (1.2% mean difference) compared with the difference between the “before” and the “after” for the same 108 candidates (21.7% mean difference). If the candidates that dropped out of the programme did this due to academic problems related to skills acquisition, this would have added more limitations to the generalization of our results. It is, however, not the case; in this case, the dropouts reflect the serious problem of brain drain from underdeveloped to more rich countries; administrative data (personal communication) from GFMP based on a questionnaire filled by the candidates before leaving shows that more than 80% left to Saudi Arabia due to economic reasons. We believe this is rather independent of the skills acquisition.

A second limitation is the use of self-evaluation as an assessment method. It is unclear how well self-assessment methods correlate with the actual skills performance when externally evaluated [28, 29]. However, self-evaluation is valuable in reflecting an individual's own confidence in performing certain skills, which is widely accepted for use as an assessment tool for further development [30–32], and, for this reason, it has been used in this study. Self-assessment is also a feasible method and may cover a big number of skills compared with other assessment methods, including objective structured clinical examination (OSCE), which allows only a small number of skills to be tested [33]. Another issue in the use of self-evaluation as a subjective assessment method is whether the increase in the total mean score from 3.23 to 3.93 (21.7%) over two years is better or poorer than expected as a result of the curriculum or if it corresponds to a significant change of phase in the Dreyfus model of skills [8, 9]. If we assume a significant association between Dreyfus' five levels of competence and our five levels of confidence, the increase found would correspond to a mean change from “competent” to close to “proficient.” “Competence” requires considerable experience [9], while level 2 (advanced beginner) does not require personal responsibility, which is clearly necessary to act as a clinical master candidate in family medicine. It seems reasonable to conclude that the achievement obtained may be relevant in both absolute change and level for a master's programme in family medicine. In his review article, Peña discusses several controversial points attached to the Dreyfus model [9]. He concludes that even if it may be an accepted and reasonable stair of competence to be adapted for medical education, it is debatable if it is based on current pedagogical and psychological knowledge and thus can explain the acquisition of clinical skills. The GFMP tried to assess competence in clinical skills both theoretically and practically during programme evaluations and exams. The results showed that candidates mostly reached their targeted level, and very few failed in formal settings (administrative data, GFMP personal communication).

It is important not to confuse competence with confidence: the first is objective and the second is subjective, and there is an interesting relationship between the two. Some students are overconfident, while others are not so confident relative to objective assessments of their skills. Both of these situations are problematic, particularly in the extreme, and should not be disregarded or minimized. The present study's scope was to show how development was reflected in candidates' confidence, and we have no data directly linking subjective confidence to objective competence.

Thirdly, a design that included a control group would have added more strength to this study. The project targeted all medical doctors in Gezira to be enrolled in the programme; the only requirement was one year of experience after graduation (internship). In addition to the newly graduated doctors after their internship and doctors from other medical disciplines, the vast majority of district practicing medical doctors (115 doctors of about 150) joined the programme. The group of district practicing doctors could have been a potential control group, but they were instead enrolled in the GFMP.

### 4.2. Implications for Practice

Family medicine is a rapidly developing and transforming discipline at the regional and worldwide levels. Unfortunately, there is scarcity of literature assessing the outcome of postgraduate training programmes [33]. The curriculum style of the GFMP master's programme was based on a modern postgraduate family medicine training approach [23] that relies heavily on information and communication technology [24] in a mixed, in-service model of training that follows a competency-based and community-centred curriculum. The curriculum design is also intended to assist in reaching an international target of high upgrading of family medicine training [7, 17]. It is therefore worthwhile to gain experience and learn from the GFMP training model and to assess its outcome. Sharing the outcomes of this experience with other family medicine training programmes is expected to contribute towards further upgrading of postgraduate training in family medicine, with emphasis on the quality of training and the training outcomes.

The study pays attention to certain issues that need further scrutiny and more research; these issues include family-medicine-related postgraduate skills and curriculum design, evaluation of training outcomes, gender and interdisciplinary variation, and correlation patterns between skills confidence and competence.

## 5. Conclusion and Recommendations

This study evaluates the impact of the GFMP training's curriculum on the candidates' own confidence in performing certain clinical tasks. Results showed a general positive progress trend with some interdisciplinary and gender variations. Further research is needed to measure the impact of the family physicians' training on the health system and patient care.

## Figures and Tables

**Table 1:** Self-assessment in performing 46 clinical skills before and after the GFMP, Gezira state, Sudan (*N* = 108). Numbers represent means for each skill. Change is the absolute difference between the "after" result and the "before" result. Ratio is the relative change calculated by the "after" result divided by the "before" result.

Clinical skills categorized in disciplines	Before	After	Change	Ratio	*P* value
*Internal medicine*	**3.56 **	**4.24**	**0.68**	1.19	**<0.001**
ECG interpretation	2.54	3.83	1.29	1.51	<0.001
ECG taking	2.28	3.45	1.17	1.51	<0.001
Management of myocardial infarction	3.49	4.30	0.81	1.23	<0.001
Management of diabetic coma	3.76	4.38	0.62	1.16	<0.001
Cardiopulmonary resuscitation	3.50	4.11	0.61	1.17	<0.001
Insulin treatment in hyperglycaemia	3.92	4.47	0.55	1.14	<0.001
Inhaler technique	3.98	4.51	0.53	1.13	<0.001
Management of tuberculosis	3.63	4.08	0.45	1.12	0.012
Venipuncture and drip start	4.26	4.63	0.37	1.08	0.003
Management of asthma	4.25	4.60	0.35	1.08	<0.001
*Laboratory skills*	**2.62**	**3.70**	**1.08**	1.41	**<0.001**
Glucose measuring	2.42	3.75	1.33	1.55	<0.001
Urine analysis	2.30	3.47	1.17	1.51	<0.001
Haemoglobin measuring	2.79	3.86	1.07	1.38	<0.001
Blood film for malaria	2.98	3.75	0.77	1.26	<0.001
*Obstetrics and gynaecology*	**3.32**	**3.84**	**0.52**	1.16	**<0.001**
IUCD insertion	2.56	3.54	0.98	1.38	<0.001
Taking cervical smear	2.73	3.59	0.86	1.32	<0.001
Normal delivery	3.51	4.02	0.51	1.20	<0.001
Acute vaginal bleeding	3.44	3.84	0.40	1.12	0.018
Vaginal examination	4.10	4.43	0.33	1.08	0.012
Caesarean section	3.06	3.39	0.33	1.11	0.004
Evacuation after abortion	3.86	4.07	0.21	1.05	0.072
*Minor surgery*	**3.90**	**4.30**	**0.40**	1.10	**<0.001**
Plaster of minor fractures	3.21	3.80	0.59	1.18	<0.001
Management of urinary retention	3.70	4.18	0.48	1.13	<0.001
Urethral catheterization	4.13	4.49	0.36	1.09	<0.001
Suturing of wounds	4.47	4.65	0.18	1.04	0.010
Abscess drainage	4.38	4.54	0.16	1.04	0.038
Stopping epistaxis	3.51	4.14	0.63	1.18	<0.001
*Major surgery*	**2.56**	**3.07**	**0.51**	1.20	**<0.001**
Acute abdomen operation	2.11	2.76	0.65	1.31	<0.001
Appendectomy operation	3.01	3.38	0.37	1.12	0.003
*Ophthalmology*	**2.34**	**3.68**	**1.34**	1.57	**<0.001**
Visual acuity	2.64	4.25	1.61	1.61	<0.001
Eye fundoscopy	2.08	3.51	1.43	1.69	<0.001
Removal of foreign body from the eye	2.51	3.73	1.22	1.49	<0.001
Management of iridocyclitis	2.14	3.21	1.07	1.50	<0.001
*Psychiatry*	**2.33**	**3.63**	**1.30**	1.56	**<0.001**
Management of major depression	2.16	3.61	1.45	1.67	<0.001
Acute psychosis	2.49	3.65	1.16	1.47	<0.001
*Other skills*					
Diagnosis of hearing loss	2.71	3.78	1.07	1.39	<0.001
Tonsillitis	4.37	4.46	0.09	1.02	0.546
Chest X-ray in infections	3.20	4.09	0.89	1.28	<0.001
X-ray interpretation in trauma	3.26	4.04	0.78	1.24	<0.001
Cholesteatoma operation	1.72	2.48	0.76	1.44	<0.001
Thoracal drainage	2.28	2.93	0.65	1.29	<0.001
Insertion of nasogastric tube	3.87	4.35	0.48	1.17	<0.001
Knee examination	3.90	4.32	0.42	1.11	<0.001
Malnutrition in children	3.99	4.41	0.42	1.11	<0.001
Stabilization of major fractures	2.71	3.24	0.53	1.20	<0.001
Measuring blood pressure	4.78	4.88	0.10	1.02	0.048
*Total for all skills (mean)*	**3.23**	**3.93**	**0.70**	1.22	**<0.001**

**Table 2:** Changes in the numbers and percentages of the master's programme candidates who were “unable” or “uncertain” when performing certain skills before and after the GFMP, Gezira state, Sudan (*N* = 108).

Sequence by change percentage	Skills	Unable or uncertain			
		Before (*n*)	After (*n*)	Change (*n*)	Change (%)
(1)	Visual acuity	46	3	43	93
(2)	ECG interpretation	41	5	36	88
(3)	Management of major depression	64	13	51	80
(4)	ECG taking	61	13	48	79
(5)	Glucose measurement	57	13	44	77
(6)	Eye fundoscopy	68	16	52	76
(7)	Acute psychosis	48	12	36	75
(8)	Haemoglobin measurement	44	11	33	75
(9)	Cardiopulmonary resuscitation	20	5	15	75
(10)	Diagnosis of hearing loss	38	10	28	73
(11)	Eye foreign body removal	53	14	39	74
(12)	Chest X-ray in infections	25	7	18	72
(13)	Urine analysis	59	18	41	69
(14)	Taking cervical smear	41	14	27	65
(15)	IUCD insertion	51	18	33	65
(16)	Blood film for malaria	32	13	19	59
(17)	Management of iridocyclitis	61	30	31	51
(18)	Caesarean section operation	25	14	11	44
(19)	Appendectomy operation	31	19	12	39
(20)	Acute abdomen operation	63	40	23	37
(21)	Stabilization of major fractures	39	25	14	36
(22)	Thoracal drainage	52	35	17	33
(23)	Cholesteatoma operation	75	52	23	31

**Table 3:** GFMP candidates' self-assessment of competences in certain patient-educational skills before and after the master's programme (*N* = 108) (scale: “yes” = 2, “sometimes” = 1, and “no” = 0).

Patient-educational skill	Before (*n*)					After (*n*)					Change of mean value	*P* value
	Yes	Sometimes	No	Missing	Mean value	Yes	Sometimes	No	Missing	Mean value		
Teaching diabetic patients how to use insulin	45	41	20	2	1.24	69	35	3	1	1.62	0.38	<0.001
Teaching women breast self-examination	31	35	39	3	0.92	59	40	9	0	1.46	0.54	<0.001
Discussing smoking cessation with patients	56	36	12	4	1.42	81	24	3	0	1.72	0.30	<0.001
Teaching mothers how to feed malnourished children	82	16	8	2	1.70	96	10	2	0	1.87	0.17	0.009

**Table 4:** Gender variation in self-assessed competences in discipline-based groups of skills among master's programme's candidates at the GFMP, Gezira state, Sudan (*N* = 108, 46 males and 62 females).

Group of skills	Before			After		
	Male	Female	*P* value	Male	Female	*P* value
Internal medicine	3.79	3.41	0.006	4.33	4.14	0.075
Laboratory skills	2.89	2.43	0.043	3.85	3.60	0.193
Minor surgery	4.24	3.62	<0.001	4.44	4.17	0.031
Major surgery	3.01	2.27	<0.001	3.41	2.74	0.001
Ophthalmology	2.47	2.27	0.243	3.82	3.56	0.110
Obstetrics and gynaecology	3.66	3.07	<0.001	4.07	3.67	0.002
Psychiatry	2.86	1.95	<0.001	3.89	3.44	0.014
All skills	3.52	3.02	<0.001	4.09	3.80	0.006

**Table 5:** Regression analyses for factors influencing change in candidates' self-evaluation of own competence in clinical skills after the master's programme. Changes in skills are shown by three different measures: residual gain score, percent change, and raw score (see Methods for details).

Variable	Residual gain score			Percent change score			Raw change score		
	*B*	Beta	*P*	*B*	Beta	*P*	*B*	Beta	*P*
Gender	−0.10	−0.09	0.44	12.4	0.25	0.04	0.27	0.21	0.08
Age	0.00	0.02	0.88	0.51	0.14	0.26	0.01	0.14	0.26
Locality	0.07	0.07	0.57	1.89	0.04	0.75	0.07	0.05	0.65
Graduating university	0.07	0.07	0.52	1.34	0.03	0.80	0.05	0.04	0.71
Clinical activity	0.00004	0.09	0.39	0.002	0.07	0.50	0.00003	0.05	0.62
Interest in family medicine	0.21	0.24	0.02	3.44	0.08	0.43	0.15	0.14	0.17

Constant	−0.951		0.06	−31.08		0.22	−0.918		0.14
Determination coefficient (*R*^2^)	0.089			0.084			0.087		
*F*-value	1.62			1.51			1.58		
All-over *P* value for model	0.15			0.18			0.16		
